# Urinary Stem Cells as Tools to Study Genetic Disease: Overview of the Literature

**DOI:** 10.3390/jcm8050627

**Published:** 2019-05-08

**Authors:** Maria Sofia Falzarano, Alessandra Ferlini

**Affiliations:** 1 UOL (Unita` Operativa Logistica) of Medical Genetics, University of Ferrara, 44121 Ferrara, Italy; 2 Neuromuscular Unit, Great Ormond Street Hospital, University College London, Bloomsbury, London WC1E 6BT, UK

**Keywords:** urinary cells, stem cells, induced pluripotent stem cells, genetic disease, tissue regeneration, differentiation

## Abstract

Urine specimens represent a novel and non-invasive approach to isolate patient-specific stem cells by easy and low-cost procedures, replacing the traditional sources (muscle/skin biopsy/adipose tissue) obtained with invasive and time-consuming methods. Urine-derived stem cells (USCs) can be used in a broad field of applications, such as regenerative medicine, cell therapy, diagnostic testing, disease modelling and drug screening. USCs are a good source of cells for generating induced pluripotent stem cells (iPSCs) and importantly, they can also be directly converted into specific cell lines. In this review, we show the features of USCs and their use as a promising in vitro model to study genetic diseases.

## 1. Introduction

Stem cells represent a precious research tool used in different research, pre-clinical and clinical studies [1]. Stem cells are undifferentiated cells with self-renewing capabilities and can differentiate into multiple cell lineages.

**Figure 1:**
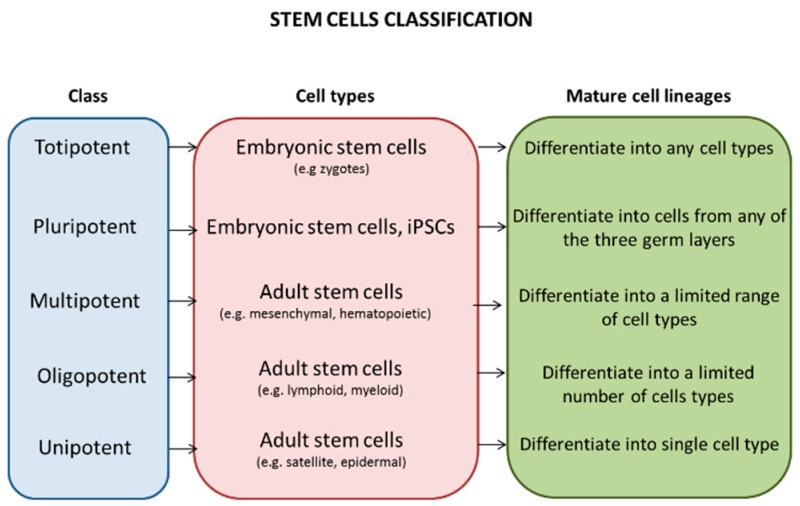
Classification of stem cells. Stem cells can be classified according to their plasticity in: Totipotent that give rise to all embryonic and extraembryonic cell lines; pluripotent that can produce all embryonic cell types; multipotent that differentiate to a great number of cell types; oligopotent that have the ability to differentiate into only a few cell lineages; and unipotent that give rise to only one specific cell type.

Based on the differentiation features, stem cells can be classified in: Pluripotent cells like the embryonic stem that can give rise to any cell types of all three embryonic lineages and grow indefinitely in culture; and multipotent or unipotent cells like the adult stem cells that can differentiate into a limited number of mature cell types [2] (Figure 1).

Despite human embryonic stem cells (hES) offering huge opportunities for new therapies in the treatment of diseases, their use has been highly discussed, because (i) they are derived from early embryos, and (ii) they activate immune responses and/or rejection.

Adult stem cells (also known as somatic stem cells) can be found after embryonic development in tissues such as brain, blood, skeletal muscles, bone marrow, adipose tissue, skin and liver. They remain in a quiescent and undifferentiated state until a disease or tissue injury activates their proliferation in order to repair the damaged tissue in which they reside and maintain tissue homeostasis.

Mesenchymal stem cells (MSCs) are an example of multipotent adult stem cells characterized by their adherence to plastic surfaces with an extensive proliferation capacity in vitro as well as in vivo. They are called mesenchymal stem cells because they can differentiate into various mesoderm-type cells such as osteoblasts, chondrocytes, myocytes, adipocytes [3].

Satellite cells (SCs) of skeletal muscle are an example of unipotent cells involved in muscle regeneration. SCs are located between the basal lamina and plasma membrane of the muscle fibres (sarcolemma) and are normally quiescent in adult muscle until a stimulus or damage takes place and activates SCs to trigger the formation of new muscle fibres [4,5].

The main issues of adult stem cells are both the limited number of cell types that can be obtained and the traditional methods that are used to harvest them, especially when paediatric patients are involved. The generation of induced pluripotent stem cells (iPSCs) provided a great opportunity to overcome the ethical issues related to the hES cells and the limitation related to the proliferation rate of adult stem cells [6].

Different reprogramming methods have been developed to convert somatic cells into iPSCs as a tool for different clinical and research applications. The iPSCs display self-renewal and pluripotency capacities and they can be generated from different cell types with various reprogramming efficiency based on the cell lines [7,8].

Despite their potential utility, some limitations regarding low reprogramming and differentiation efficiency, long-term manipulation, tumorigenicity and invasive procedures required for collecting most of them, prevented the use of iPSCs in the clinical application [9].

Ideally, the best source of cells should be obtained universally from healthy subjects or patients of any age, gender and ethnic origin by non-invasive, simple and low-cost procedures [10].

A subpopulation of urine-derived stem cells (USCs), recently identified in urine samples, is considered a promising cell resource for generating autologous stem cells to model disease because of their non-invasive, easy and low expense methods of isolation. USCs can be isolated from human urine as well as animal urine and show similar features of mesenchymal stem cells, which can potentially differentiate towards osteocytes, chondrocytes, adipocytes, myocytes and endothelial cells [11].

## 2. Isolation and Characterisation of USCs

The collection and isolation of USCs from urine samples is an easy, quick, reproducible and optimised method that allows urine cells to be obtained from healthy donors as well as from patients.

**Figure 2:**
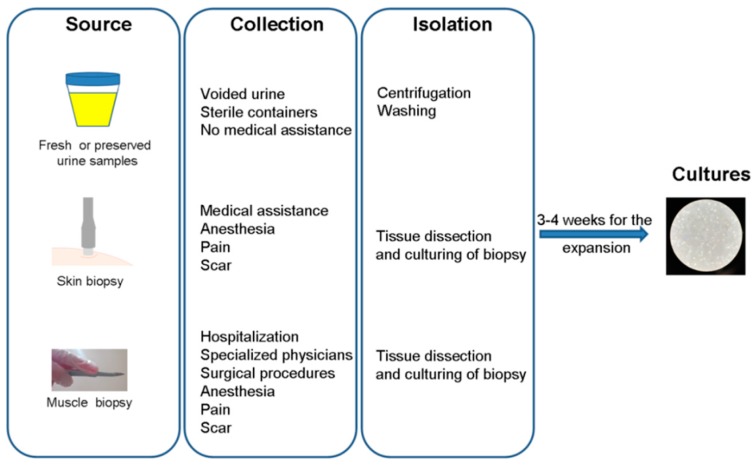
Schematic overview of three methodological procedures for cell isolation from different sources.

Several authors have described the isolation of USCs, albeit with slight differences mainly related to the composition of the medium. Briefly, the general protocol includes the collection of urine (high urine amounts give higher yields) followed by centrifugation and washing with phosphate buffered saline (PBS). The cells are then cultured with medium in gelatin-coated 24-well plates [10,11] (Figure 2).

A critical step is the preservation of the urine after its collection, since the USCs can only survive for a few hours without any preservation. Ideally, the USCs should be isolated immediately or within 4 h (from refrigerated urine) after the collection [12]. Lang and collaborators demonstrated that it is also possible to isolate USCs from 24-hour preserved urine using specific medium (solutions with 0.5%–10% serum were the best environments for USCs during cold storage), and that the USCs maintain the original stem cell properties such as shape, expression of MSCs surface markers, high telomerase activity and normal karyotypes [13]. This method makes it easier for the distribution of urine samples from clinical centres where cell isolation it is not available to sites where the isolation and cultures of USCs can be done. This is a great opportunity for patients affected with diseases that hamper the mobility of their transfer.

As described above, the simple procedure requires only centrifugation, media and coating, and for this reason the cost is very low (less than 80€ per sample), especially compared to a biopsy that requires hospitalization, specialized physicians and surgical procedures [14].

The voided urine contains a heterogeneous population of cells and most of them are differentiated cells that do not attach to the plastic plate. Only 0.1% cells in urine can attach in culture, although they do not expand further after subculture, unlike the subpopulation of USCs [10].

**Figure 3:**
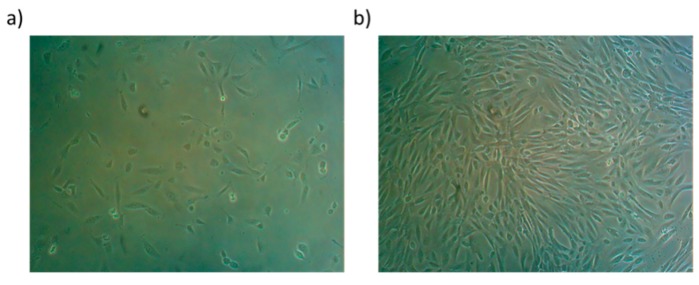
Cell morphology of early adherent urine-derived stem cells (USCs) 15 days (**a**, P1) and 21 days (**b**, P4) after isolation from urine specimens.

Small colonies usually appear 7–15 days after plating (Figure 3a) and they possess a high proliferative capacity (Figure 3b) with telomerase activity, long telomeres and low levels of senescence-associated protein and apoptotic markers [15,16,17].

The USCs carry a diploid set of chromosomes with normal karyotype also after several passages and do not form teratomas in vivo [17].

The USCs display expressions of MSCs surface markers such as CD166, CD105, CD13, CD54, CD73, CD90, CD29 and CD49a with absence of hematopoietic surface markers CD14, CD34, CD45, CD31, CD184 and HLA-DR [15,18,19,20]. In addition, the USCs are positive for pericyte (CD146) endothelial (vWF), epithelial, smooth muscle (α-SMA, Desmin) and interstitial (c-Kit) markers [15,18].

Cultured USCs can differentiate into osteogenic, chondrogenic, adipogenic, myogenic, endothelial (mesoderm) and neurogenic (ectoderm) lines in vitro [16,17,21].

Therefore, the morphology, phenotype and pluripotency features expressed by USCs resemble the currently accepted biological characteristics of MSCs [21,22].

The possible origin of voided USCs may be the kidney, more specifically the glomerulus. Indeed, cells obtained from female patients who received a sex-mismatched kidney transplant showed the presence of the Y chromosome and expressed normal kidney cell gene and protein markers [17]. In addition, voided USCs display similar morphology, cell phenotypes, growth patterns and differentiation capacity of the USCs obtained from the upper urinary tract, suggesting they originate from the upper urinary tract. Moreover, the USCs express CD146+/CD31− markers with comparable expression levels of parietal cells and podocytes in glomerulus, while renal tubule epithelial cells, bladder and ureter urothelial and smooth muscle cells are not detected [16].

## 3. Application of Urine-Derived Stem Cells in Genetic Diseases

Nowadays, there is growing interest in using urine as an in vitro model to study disease mechanisms, to identify new biomarkers, to test compounds, and to use gene-editing approaches. Investigating the molecular mechanisms that underlie the pathology is essential especially for diseases where the genetic bases remain unknown or no cures are currently available.

Reprogramming techniques are often used to generate induced pluripotent stem cells (iPSCs) from patient-derived urine cells since they possess a high level of reprogramming efficiency, up to 4% [18]. Direct reprogramming is another option to generate patient-specific cell lineages without the iPSCs step, in order to reduce both costs and time required to obtain specific cell types and to enhance the efficiency of the method.

In this section, the authors describe the potential application of urine-derived cells for studying genetic diseases using native urine-derived stem cells (Section 3.1), direct reprogrammed urine cells (Section 3.2), and urine-induced pluripotent stem cells (Section 3.3) (Table 1).

### 3.1. Urine-Derived Cells as a Model to Study Genetic Diseases

For the first time, Slaats and colleagues demonstrated the potential employment of urine-derived cells as a cellular in vitro model for diagnosis and pathophysiologic evaluation of patients with Fabry disease (FD). Fabry disease is an X-linked lysosomal disorder caused by mutations in the alpha-galactosidase A (*GLA*) gene. The mutation causes a deficiency of the enzyme alpha-galactosidase A (α-GalA), resulting in excessive deposition of globotriaosylceramide (Gb3) in enlarged lysosomes of various cell types [23]. FD is a multi-organ disease and the kidney, heart and brain are predominantly involved. The authors showed that USCs from FD patients can be used to measure α-GalA enzyme activity, Gb3 levels, RNA levels and proteomic alterations. They found that the urine cells obtained from FD patients exhibit a significant decrease of α-GalA enzyme activity compared to controls as well as the accumulation of Gb3. Since the Gb3 measurements in urine resulted to be very inconsistent, the possibility of measuring the enzyme activity in urinary cells could help in the Fabry disease diagnosis and the monitoring of therapies using a non-invasive material. In addition, the proteomic analysis showed the upregulation of lysosomal proteins in patient samples compared to controls, due to α-GalA impairment, and these findings could be used as a first step in evaluating prognostic biomarkers in patients’ urine [24].

Inherited epidermolysis bullosa (EB) is a group of heterogeneous genetic diseases caused by mutations in at least 17 genes that encode for intracellular, transmembrane or extracellular proteins. EB is characterised by fragile skin (formation of blisters, erosions and wounds) and mucous membranes [25]. Currently, there is no cure for EB, although progress has been made in testing novel treatments in gene therapy, cell therapy and bone marrow transplantation fields [26]. Stem cells play a central role in regenerative medicine and the isolation of them from patients affected by severe genetic skin diseases like EB could be extremely painful and not feasible. Therefore, Schosserer and collaborators isolated and characterised USCs from patients with EB. They confirmed the expression of typical markers of MSC, the ability to differentiate into osteogenic, chondrogenic and adipogenic cell lines, and the immune-modulatory properties. These results suggest a promising non-invasive way to obtain stem cells for therapeutic approaches and to improve medical conditions of patients with EB [22].

Urine cell cultures were also established from spinal muscular atrophy (SMA) patients. SMA is a recessive disorder caused by the homozygous deletion of motor neuron 1 gene (*SMN1*). The application of patient-derived urine cells has been tested in order to replace the muscle or skin biopsy procedures that are considered unacceptable for young patients with SMA. Urine cells derived from SMA patients carried *SMN* gene mutations and showed low levels of SMN protein compared with controls. The treatment with both histone deacetylase inhibitors and morpholino modified antisense oligo upregulated the levels of SMN, indicating that SMA patient-derived urine cells may be used as a tool for molecular studies and for screening of potential compounds and drugs to treat SMA [27,28].

Lastly, human urine-derived cells can be used as an innovative tool for modelling of genetic kidney disorders and to complement the diagnosis of inherited renal diseases through the study of the functional effects caused by potentially pathogenic mutations with unknown significance [29].

### 3.2. Direct Reprogramming of Urine-Derived Cells

The direct reprogramming of urine cells in myogenic lineage has been applied to study muscle diseases. Two works published in 2016 showed that USCs can be efficiently reprogrammed into myogenic cells through the viral delivery of the muscle transcription factor MyoD [15,30]. This strategy was successfully applied to generate myogenic cells from: (i) USCs derived from healthy donors, (ii) two patients with limb girdle muscular dystrophy type 2C (LGMD2C), which results from loss of function mutations in the gene encoding γ-sarcoglycan, (iii) two Duchenne muscular dystrophy (DMD) patients with frameshift deletions of exons 46–47 and exon 45, respectively. Both works demonstrated the full-length DMD transcript expression in reprogrammed healthy MyoD-USCs and the truncated DMD transcript expression in the reprogrammed MyoD patient cells. In addition, Falzarano et al. revealed the presence of dystrophin transcript also in native USCs, not MyoD reprogrammed, from both controls and a DMD patient. As expected, dystrophin protein was detected only in reprogrammed control cells but not in DMD patient cells, due to the frameshift mutations that lead to the absence of dystrophin protein. In order to determine the feasibility of this model to test drugs and new therapeutic approaches, the MyoD-USCs were also used to test the efficacy of both antisense oligoribonucletides (AONs) treatment and CRISPR/Cas9 editing. Both strategies demonstrated the ability to induce exon skipping with AON and to edit the genome with CRISPR/Cas9 in urine-derived cells. Thus, the MyoD reprogramming in urine cells is able to recapitulate the primary LGMD and DMD phenotypes in vitro [15,30].

A recent study reported a novel direct-reprogramming system of human USCs into myotubes. The authors developed a retroviral doxycycline (Dox)-regulated inducible MYOD1 expression system for the selection of cells by antibiotic (puromycin) and for the regulation of cell proliferation/differentiation after MyoD transduction. Moreover, the 3-deazaneplanocin A hydrochloride (DZNep), a histone methyltransferase inhibitor, was used to successfully promote the differentiation of USCs into myotubes. Lastly, the authors assessed if the model was suitable for exon-skipping studies in USCs derived from DMD patients. They showed that AONs targeting DMD exons, including 44, 50, 51 and 55, induced the skipping of the specific targeted exon [31].

In this context, the USCs can be used as an alternative source to muscle and skin biopsies to explore DNA, RNA and protein profiles for diagnostic and research purposes in neuromuscular diseases.

A different approach for the differentiation of USCs in skeletal myogenic lineage cells and additionally in endothelial cells, is the use of alginate microbeads loaded with specific growth factors. It has been demonstrated that the release of a combination of growth factors that induce myogenesis, angiogenesis and innervation from microbeads can efficiently give rise to the differentiation of USCs in muscular and endothelial cells in vivo, enhancing the revascularization and innervation and stimulating resident cell growth [32].

The human urine cells can also be directly converted into functional neurons using a combination of the five specific transcriptional factors Ascl1, Brn2, NeuroD, c-Myc and Mytl1, associated with various neurotrophic factors. This method was applied to convert urine-derived cells from both healthy donors and patients with Wilson’s disease (WD), a genetic disorder caused by mutations in the *ATP7B* gene. The generation of neurons from non-neural lineages is a significant progress for the studies of neurological diseases [33].

### 3.3. Urine-Derived Induced Pluripotent Stem Cells (iPSCs) to Study Genetic Diseases

Hemophilia A (HA) is an X-chromosome-linked, recessive, severe genetic disorder caused by the deficiency or defective of factor VIII (FVIII), a clotting protein. Currently, two treatments are available for HA, fixed dose FVIII prophylaxis or factor replacement therapies.

Although these therapies have been very successful, some limitations remain such as efficacy, cost, availability, and side effects, indicating the need for new drugs and treatments. The most commonly used models to study disease mechanisms and drug screening of HA were animals, including mice, dogs and pigs, but, unfortunately, they did not mirror the human pathophysiology [34]. Jia and collaborators provided a new in vitro model based on HA patient-specific iPSCs and iPSC-derived hepatocyte-like cells [35]. They generated iPSCs from HA patients’ urine by integration-free episomal vectors and then differentiated the iPSCs in functional hepatocyte-like cells that recapitulated the phenotype of HA disease in vitro. This model will be useful for the screening of new drugs and for the personalized treatment and cell therapy of HA patients [35].

Human urine-derived induced pluripotent stem cells (UhiPSCs), differentiated into hepatocyte-like cells, were also validated as a tool to model PCSK9-mediated hypercholesterolemia. Familial hypercholesterolemia (FH) is a major risk factor for coronary artery disease and, despite the progress of pharmacological therapy, FH remains one of the main causes of mortality and disability. FH is primarily caused by mutations of three genes: LDL receptor (*Ldlr*), apolipoprotein B *(apoB)* and the proprotein convertase subtilisin/kexin 9 (*PCSK9*). Designing more efficient drugs is needed to overcome the limitations of standard therapy. The urine specimens provided an attractive and useful source of uhiPSC-derived hepatocytes for studying in vitro *PCSK9* mutations and function, and for the identification and validation of new PCSK9 inhibitors or modulators [36,37].

UhiPSCs were also obtained from a patient with phenylketonuria (PKU). PKU is a biochemical genetic disorder characterized by mutations in the phenylalanine hydroxylase (*PAH*) gene that catalyses the conversion of phenylalanine (Phe) to tyrosine. The defect causes accumulation of phenylalanine in the blood and brain, producing intellectual disability and other neurologic features [38]. A non-invasive and integration-free approach has been developed to generate iPSCs from PKU urine-derived cells that were differentiated into neurons. This model could overcome the issues associated to both the lack of an appropriate disease model and the difficulty in obtaining neural cells. Moreover, the differentiation of iPSCs into osteoblasts and osteoclasts could allow the study of mechanisms underlying bone impairment in PKU in order to identify new targets and treatments [39].

The generation of iPSCs from urine samples is considered a promising tool for application in down syndrome (DS) studies, a genetic disorder caused by trisomy 21 (TS21). T21-iPSCs have been generated from patients with DS, and glutamatergic neurons and cardiomyocytes were obtained.

The application of iPSCs in modelling DS and other neurodevelopmental and neurodegenerative disorders improve the possibility to have human cell-based models for drug screening [40].

Machado-Joseph disease (MJD), also known as Spinocerebellar ataxia type 3 (SCA3), is an inherited neurodegenerative disease caused by a CAG repeat expansion in the region of the *ATXN3* gene and associated with severe clinical phenotype and premature death. Urine cells of a SCA3 patient were successfully reprogrammed in iPSCs using the Sendai virus delivery system, providing a robust model for studying the SCA3 pathogenesis, drug testing and gene therapy research [41].

UhiPSCs lines are also able to model the paroxysmal kinesigenic dyskinesia (PKD), a monogenic movement disorder with autosomal dominant inheritance. Proline-rich transmembrane protein 2 gene (*PRRT2*) is the causative gene of PKD, although the function of the protein produced from this gene is unknown. UhiPSCs, obtained from a patient with PKD, maintained the disease specific mutation, showed lower PRRT2 levels compared with the control and were successfully differentiated into glutamatergic, dopaminergic and spinal motor neurons. Therefore, PKD-UhiPSCs could be a valuable model to investigate the pathogenic mechanisms of PKD [42].

Furthermore, the UhiPSC-based approach has been the key for the generation of cell lines dependent on patients’ genetic backgrounds in the field of cardiac diseases. Despite animal models having improved both the understanding of the genetics underlying inherited cardiovascular disorders and the development of treatments, the limitations due to interspecies differences have required new reliable experimental models. Obtaining human cardiomyocytes is extremely difficult and the feasibility of generating functional cardiomyocytes from UhiPSCs by a non-invasive method provides a solid platform for further studies.

UhiPSCs from a patient with ventricular septal defects (VSDs) and heart failure (HF) showed the retention of the mutation in the ryanodine receptor type 2 (*RyR2*), a gene that plays an important role in HF progression. In addition, the UhiPSCs were directly differentiated using small molecules in cardiomyocytes that showed spontaneous contraction and strong expression of cardiac-specific proteins and genes [43].

Cells obtained from urine samples of a patient with a type 2 long QT (LQT2) syndrome, carrying ion channel mutations, were reprogrammed in UhiPSCs and then differentiated into cardiomyocytes in order to assess their benefit for studying cardiac arrhythmia phenotypes. UhiPS-derived cardiomyocytes showed the expression of specific atrial/ventricular myofilament proteins and ion channels, and they appeared electrically functional. This work demonstrates that UhiPS cells derived from patients with ion channel mutations can be used as a model to differentiate in functional cardiomyocytes that recapitulate cardiac arrhythmia phenotypes [20].

## 4. Summary and Conclusions

The cells derived from human urine samples possess unique features for the generation of experimental in vitro models. They can be collected by completely non-invasive procedures that can be applied universally to any gender, race and age. Importantly, there is a subpopulation of urine derived cells called urine-derived stem cells (USCs) that expresses markers of stemness, such as mesenchymal surface markers, high telomerase activity and reprogramming factors. The USCs provide a useful model for human diseases, including genetic disorders. The authors mentioned some examples of applications of both patient-specific native USCs and UhiPSCs to study the mechanisms of genetic disease. We described the feasibility of inducing the differentiation of urine-derived cells into skeletal muscle, cardiac muscle, neuronal and hepatocytes cells and therefore, their ability to recapitulate the muscular, cardiac, metabolic, neurodevelopmental and neurodegenerative disorders.

Urine cells can replace the MSC obtained from tissues via invasive ways and they can be employed as a preservation method for both patient and healthy donor cells for the generation of a bio-bank for research, therapy and transplantation applications [14].

Further studies are needed to better characterize the USCs model in clinical practice, to automate the urine cell cultures and to make the UhiPSCs technology more scalable, even if a recent study demonstrated the high-efficiency reprogramming of urine derived cells in UhiPSCs with microfluidics [44].

In summary, the use of urine-derived cells offers a novel strategy for obtaining quantitative and qualitative cells to model and study the molecular mechanisms of diseases, and they can be considered a useful approach to further advance the field of regenerative technologies, including the creation of organoid models for improving personalized therapy in renal diseases [45].

## Figures and Tables

**Table 1:** Urine-derived cells can be used to generate differentiated cells for modelling genetic diseases.

Cellular Model	Genetic Disease
Native urine-derived stem cells	Fabry disease Inherited epidermolysis bullosa Spinal muscular atrophy Genetic kidney disorders
Direct reprogrammed urine cells (MyoD, alginate microbeads-specific growth factors, transcriptional factors-neurotrophic factors)	Limb Girdle Muscular Dystrophy Duchenne Muscular Dystrophy Wilson’s disease
Urine-induced pluripotent stem cells (iPSCs)	Hemophilia A Familial hypercholesterolemia Phenylketonuria Down syndrome Spinocerebellar ataxia Paroxysmal kinesigenic dyskinesia Cardiac diseases
